# Double-Network Hydrogel Films Based on Cellulose Derivatives and κ-Carrageenan with Enhanced Mechanical Strength and Superabsorbent Properties

**DOI:** 10.3390/gels9010020

**Published:** 2022-12-27

**Authors:** Jiseon Kang, Seok Il Yun

**Affiliations:** Department of Chemical Engineering and Materials Science, College of Biochemical Engineering, Sangmyung University, Seoul 03016, Republic of Korea

**Keywords:** double network (DN) hydrogels, carboxymethyl cellulose, κ-carrageenan, hydrox-yethyl cellulose, superabsorbent

## Abstract

Covalently crosslinked sodium carboxymethyl cellulose (CMC)–hydroxyethyl cellulose (HEC) hydrogel films were prepared using citric acid (CA) as the crosslinking agent. Thereafter, the physically crosslinked κ-carrageenan (κ-CG) polymer was introduced into the CMC–HEC hydrogel structure, yielding κ-CG/CMC–HEC double network (DN) hydrogels. The κ-CG physical network provided sacrificial bonding, which effectively dissipated the stretching energy, resulting in an increase in the tensile modulus, tensile strength, and fracture energy of the DN hydrogels by 459%, 305%, and 398%, respectively, compared with those of the CMC–HEC single network (SN) hydrogel. The dried hydrogels exhibited excellent water absorbency with a maximum water-absorption capacity of 66 g/g in distilled water. Compared with the dried covalent SN gel, the dried DN hydrogels exhibited enhanced absorbency under load, attributed to their improved mechanical properties. The water-absorption capacities and kinetics were dependent on the size of the dried gel and the pH of the water.

## 1. Introduction

Hydrogels consisting of 3D networks and a large amount of water have been used in a variety of fields, including biomedical science, food science, superabsorbent chemistry, water treatment, and bioelectronics [1,2,3,4,5]. However, most hydrogels fracture easily, even under mild loading conditions, because of their poor mechanical properties. One of the strategies for improving their mechanical properties is to design two different networks that interpenetrate to form an interpenetrating network hydrogel. In particular, a network with sacrificial bonds could be interwound into another covalently loosely linked network. In this regard, the covalent bonds build the ductile primary structure and maintain the integrity of the materials. Conversely, the sacrificial bonds, which constitute the rigid and brittle network, damage upon stretching to dissipate energy and impart toughness to the materials via crack-propagation resistance. These special interpenetrating network hydrogels formed from two contrasting networks, referred to as double-network (DN) gels, have been shown to display outstanding mechanical properties, including high strength and toughness [6,7,8,9,10]. The formation of a sacrificial network in the DN gels has often been achieved by the physical crosslinking of natural polymers. The excellent mechanical strength and toughness of these hybrid DN gels originate from the synergetic effect of the two contrasting network structures. For example, upon deformation of DN gels containing κ-carrageenan (κ-CG), the rigid and brittle κ-CG network continuously fractures by unzipping the double-helical aggregates and pulling out the κ-CG chains from the double helices that efficiently dissipate energy [11,12,13]. When a DN gel is stretched, the long ductile covalent network bridges the crack and, thus, imparts stretchability to the DN gels. 

**Figure 1:**
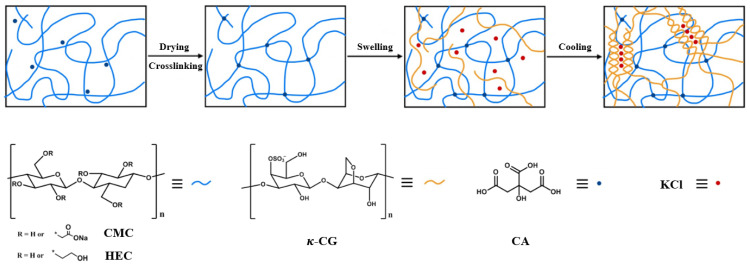
Schematic illustration of the synthesis of the DN hydrogel.

To date, polyacrylamide hydrogels are one of the most investigated covalent second networks employed in DN gels. Although polyacrylamide is widely used in biomedical applications, its monomer, acrylamide, exhibits high toxicity [14]. The use of toxic chemical crosslinking agents in covalently linked networks compromises the biocompatibility of DN hydrogels. Herein, we report DN gels composed of fully naturally derived polymers and crosslinking agents. Hydrogels based on cellulose derivatives, such as carboxymethyl cellulose (CMC) and hydroxyethyl cellulose (HEC), have been extensively utilized for a wide variety of applications because of their natural abundance, biocompatibility, and biodegradability [15,16,17,18,19]. CMC is an ether derivative of cellulose in which the H atoms of the hydroxyl groups are replaced by carboxymethyl groups (–CH_2_COOH). It is often applied in its sodium-salt form, Na–CMC, and it exhibits high pH sensitivity. CMC tends to form intramolecular crosslinks rather than intermolecular crosslinks. It has been used along with HEC, whose introduction avoids the formation of intramolecular crosslinks [15,16,17,18,19]. Previously, covalently crosslinked CMC–HEC hydrogels were successfully prepared with citric acid (CA) as the crosslinking agent [15,16,17,18,19]. In this study, covalently crosslinked CMC–HEC hydrogel films were successfully prepared with CA as the nontoxic crosslinking agent. Thereafter, the covalently crosslinked CMC–HEC dried film was immersed and swelled in a hot solution of κ-CG and potassium chloride (KCl). Due to the high swelling capacity of the cellulose network, the κ-CG slowly diffused into the cellulose network, resulting in a significant volume expansion of the CMC–HEC network. Upon cooling to room temperature, a coil-to-helix structural transition and subsequent sol–gel phase transition of κ-CG occurred, forming a “rigid and brittle” network entangled with the covalently crosslinked CMC–HEC network. Thus, DN hydrogels comprising interpenetrating networks of κ-CG and CMC–HEC were successfully obtained, as shown in Figure 1. This work aims to obtain hydrogels with improved mechanical properties. This is achieved by synergetic reinforcement using the covalently crosslinked CMC–HEC and the physically crosslinked κ-CG, exploiting the toughening mechanism of DN gels. Although the hydrogels of covalently crosslinked CMC combined with other biological polymers, such as chitosan, have been studied to improve the mechanical properties and functionalities of the hybrid gels, the construction of all-natural DN gels based on covalently crosslinked CMC/κ-CG has not been reported [20,21,22].

The swelling behavior is one of the most significant features of hydrogels. A superabsorbent polymer (SAP) is a loosely crosslinked hydrogel whose 3D chain network swells to absorb and retain water up to a hundred times its weight. The hydrogels’ ability to absorb water is attributed to the constituent hydrophilic functional groups, including –OH, –CONH–, –CONH_2_, –COOH, and –SO_3_H, attached to their carbon polymer chains. The super absorbency of CA-crosslinked CMC hydrogels has been reported, which is attributed to the electrostatic repulsion between the COOH groups of CMC and CA [23,24]. SAPs are mainly applied in hygiene goods (e.g., disposable diapers and sanitary products), sensing devices (e.g., biosensors), and agricultural products (e.g., soil additives) [3]. Recently, many researchers have attempted to utilize biomass-based natural polymers in SAPs because of their biocompatibility, biodegradability, nontoxicity, and high abundance [25,26]. The SAP synthesis route for natural polymers mostly involves the graft copolymerization of vinyl monomers or the incorporation of reinforcing particles onto the natural polymer network to enhance both the mechanical strength and water absorbency [3,25,26]. A few attempts to use interpenetrating or DN gel structures to prepare SAPs have been reported [27]. In this study, we experimented on dried κ-CG/CMC–HEC DN gels to obtain environmentally friendly superabsorbent composites with enhanced water absorbency and a high water-retention capacity. The effects of the gel size and composition on the absorption capacities and kinetics were investigated.

## 2. Results and Discussion

### 2.1. Preparation and Physicochemical Characterization of the CMC–HEC Hydrogels

**Figure 2:**
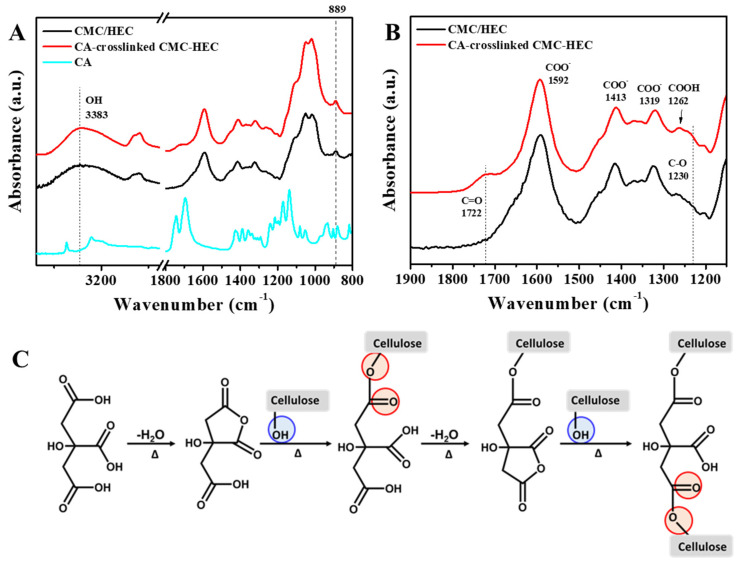
FTIR spectra of CMC/HEC, CA-crosslinked CMC–HEC, and CA in the ranges of (**A**) 3750–800 cm^−1^ and (**B**) 1900–1150 cm^−1^. (**C**) Scheme of the crosslinking mechanism of cellulose with CA.

FTIR spectroscopy was conducted to monitor the crosslinking of CMC and HEC with CA as the agent. Figure 2A,B compare the FTIR spectra of the CA-crosslinked and non-crosslinked CMC/HEC films. Both samples exhibited carboxylate bands (COO^−^) at 1592, 1413, and 1319 cm^−1^ and a carboxylic acid (COOH) band at 1262 cm^−1^ [28,29]. The cross-linking between CA and cellulose was proposed to occur via the molecular dehydration of the acid, followed by esterification (Figure 2C). Indeed, for the crosslinked films, a new band was observed at 1722 cm^−1^, which was assigned to the stretching mode of the C=O ester bond expected to appear at higher frequencies than those for carboxylic acids. A change in the relative intensity of the OH peak at approximately 3400–3200 cm^−1^ is observed, which can be estimated by the ratio of the absorbance at 3383 cm^−1^ (A3383), corresponding to the stretching vibration of the OH group that forms hydrogen bonding, to the reference band at 889 cm^−1^ (A889), related to the β1-4 glycoside bond [28,29]. Table 1 shows the depletion of the hydroxyl groups of CMC–HEC during the crosslinking; this is caused by the formation of ester bonds via the chemical reaction with CA, which is consistent with previous reports. In addition, the increased vibration at 1230 cm^−1^ for the crosslinked hydrogels was ascribed to the C–O group of the formed esters. 

**Figure 3:**
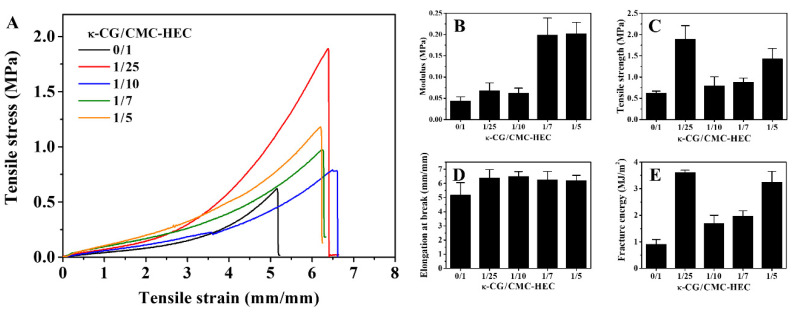
(**A**) Tensile stress–strain curves and (**B**–**E**) the corresponding mechanical parameters of the κ-CG/CMC–HEC hydrogels with different compositions.

The effect of the weight ratio of κ-CG to CMC–HEC on the tensile stress–strain curves of the DN gels was investigated (Figure 3A) at a fixed crosslinking density of CMC–HEC (C_CA_ = 4%), and the corresponding mechanical properties are shown in Figure 3B–E. Typically, with the incorporation of the κ-CG network into the CMC–HEC hydrogels, the tensile strength, modulus, and toughness of the hydrogels significantly increased. The tensile strength and fracture toughness increased from 0.62 to 1.89 MPa and 0.91 to 3.62 MJ/m^3^, respectively, when the κ-CG/CMC–HEC ratio increased from 0/1 to 1/25. Notably, pure κ-CG hydrogels are easily broken into fragments under a moderate pressure below 5 kPa (not shown). The higher strength and toughness of the DN gels compared with those of the CMC–HEC hydrogels may be ascribed to the existence of a synergistic effect between the covalently crosslinked CMC–HEC network and the physically crosslinked κ-CG network. When the κ-CG proportion was further increased to 1/7 and 1/5, the tensile modulus increased significantly to 0.199 and 0.202, respectively, although a decrease in the tensile strength was observed. The result shows that a relatively low concentration of the physical network substantially improves both the tensile strength and fracture energy, although the modulus increase is marginal. A high concentration of the physical network leads to a considerable increase in the modulus; however, this occurs at the cost of the tensile strength and fracture energy.

**Figure 4:**
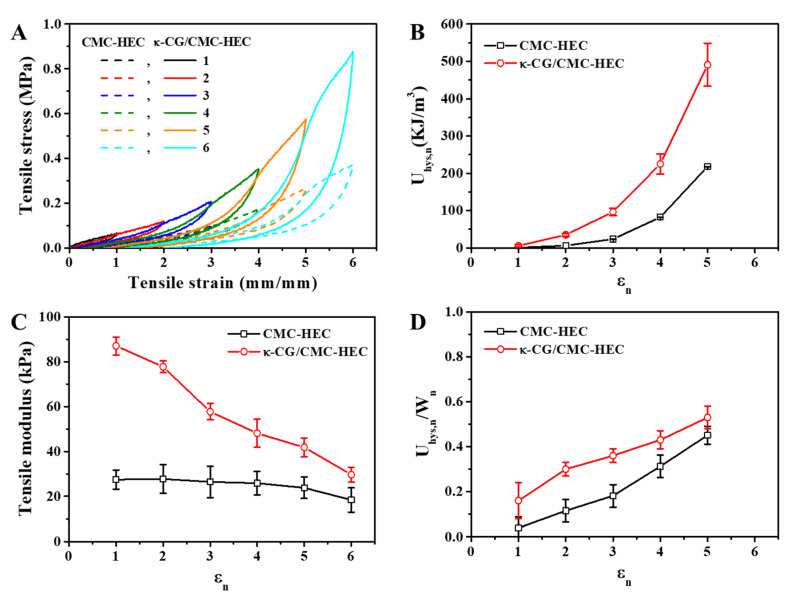
(**A**) Successive cyclic tensile loading–unloading curves of the CMC–HEC SN and κ-CG/CMC–HEC (1/25) DN hydrogels with increasing ε_n_ and without any resting time between consecutive loading cycles. The dependence of (**B**) U_hys, n_, (**C**) the tensile modulus, and (**D**) U_hys, n_/W_n_ on ε_n_ is shown.

The excellent mechanical performances, particularly the high toughness, of the DN gels are closely related to the effective energy dissipation, which can be demonstrated by the hysteresis of the loading–unloading curves of the hydrogels stretched to different maximum strain (*ε_n_*) values. As shown in Figure 4A, the hysteresis loops developed in the stress–strain curves were observed for both the CMC–HEC covalent single-network (SN) gel and the κ-CG/CMC–HEC DN hybrid gel. For both the SN and DN hydrogels, the (n + 1)^th^ loading curves are always higher than the n^th^ unloading curves, probably because of the self-recovery of the dissociated physical bonding. The energy dissipated during the n^th^ cycle was calculated as follows [30,31]:

(1) $$\Delta U_{\mathrm{hys},\;n}=\int_{0}^{\varepsilon_{n}}\sigma_{n}d\varepsilon -\int_{0}^{\varepsilon_{n}}\sigma_{n+1}d\varepsilon$$

The total accumulated energy dissipated from the 1st to the n^th^ loading is expressed as follows:

(2) $$U_{\mathrm{hys},\;n}=\overset{n}{\underset{i=1}{\sum }}\Delta U_{\mathrm{hys},\;i}$$

The work (W_n_) required to stretch the hydrogel to ε_n_ is equivalent to the total area below the stress–strain curve, as follows:

(3) $$W_{n}={\;U}_{\mathrm{hys},\;n}+\int_{0}^{\varepsilon_{n}}\sigma_{n+1}d\varepsilon$$

When the DN gel was stretched under high strain, it was more irreversibly elongated and underwent increased U_hys, n_ with increasing ε_n_ (Figure 4B). Compared with the covalent SN hydrogel, the DN hybrid hydrogel demonstrated larger hysteresis (Figure 4A), resulting in larger U_hys, n_ values (Figure 4B). This result indicates that the breakage of the physical bonds in the κ-CG networks, i.e., “sacrificial bonds,” facilitated the absorption of the deformation energy. It is well known that the physical network of the κ-CG assembly fractures gradually during the whole deformation process and, thus, provides a sacrificial network in the hybrid DN gels [11,12,13]. The hysteresis loops and resulting U_hys, n_ developed for the covalent SN hydrogel suggested the coexistence of covalent and physical crosslinkages, including chain entanglement and hydrogen bonding, between the CMC and HEC chains. With the increase in the degree of internal irreversible fracturing, the elastic density of the DN gel effectively decreases compared to the case with the SN gel, as evidenced by the decrease in the tensile modulus with increasing ε_n_ during the successive loading and unloading cycles (Figure 4C). The efficiency of energy dissipation can be further quantified using the U_hys, n_/W_n_ ratio, where W_n_ is the work performed by the extension, and the U_hys, n_/W_n_ ratio represents the fraction of the irreversible work in the total extension work due to the internal fracture. Figure 4D illustrates the relationship between U_hys, n_/W_n_ and ε_n_ for the DN gel. An increase in ε_n_ leads to a continuous increase in U_hys, n_/W_n_. This fracturing behavior with the continuous increase in U_hys, n_/W_n_ with ε_n_ highlights the gradual fracture of the physically crosslinked assemblies of long natural polymers; such as κ-CG and agar; via progressive molecular unzipping and chain pulling-out mechanisms; which provides a sacrificial first network in the hybrid DN gels [10,12,32]. 

**Figure 5:**
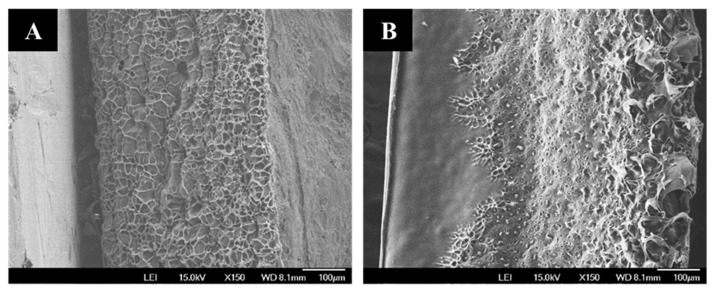
Cross-sectional SEM images of the freeze-dried hydrogels of (**A**) CMC–HEC SN and (**B**) κ-CG/CMC–HEC DN (1/25).

As shown in the SEM images (Figure 5A), the freeze-dried CMC–HEC SN hydrogels exhibited uniform morphology with both closed and open-pore structures. Conversely, the freeze-dried DN hydrogel (Figure 5B) exhibited a less porous morphology, indicating that κ-CG penetrated and filled the pores of the CMC–HEC network during the swelling process in the κ-CG solutions. 

### 2.2. Water-Absorbency Properties of the As-Prepared Dried DN Hydrogels 

**Figure 6:**
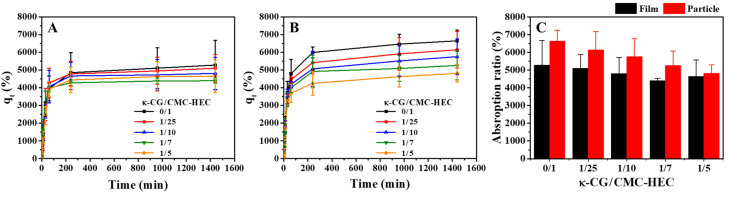
Effect of the absorption time on the water-absorption capacity of the hydrogel (**A**) films and (**B**) particles with different compositions in deionized water (pH 7) at 25 °C. (**C**) Effect of the κ-CG content on the maximum water-absorption capacities of the hydrogel films and particles in deionized water (pH 7) at 25 °C.

To compare the effect of size on the absorption capacity, the hydrogels were cut into 30 × 30 mm^2^ (film) and 10 × 10 mm^2^ (particle) pieces. The water-absorption capacities of the dried hydrogel films and particles with different compositions are plotted as a function of the absorption time in Figure 6A,B, respectively. During the initial stage before equilibrium, the absorption rates of water (pH 7) were relatively high. The maximum water-absorption capacities of the dried hydrogel films and particles with different compositions are summarized in Figure 6C. Compared with the dried film, the dried particles exhibited a higher absorption capacity, attributed to their increased interface area for water absorption. With increasing the κ-CG content, the water-absorption capacities of both the film and particle decreased. The less porous morphology of the DN gel network compared with that of the CMC–HEC network observed by SEM (Figure 5) could be responsible for the decrease in the absorption capacity with increasing κ-CG content. 

**Figure 7:**
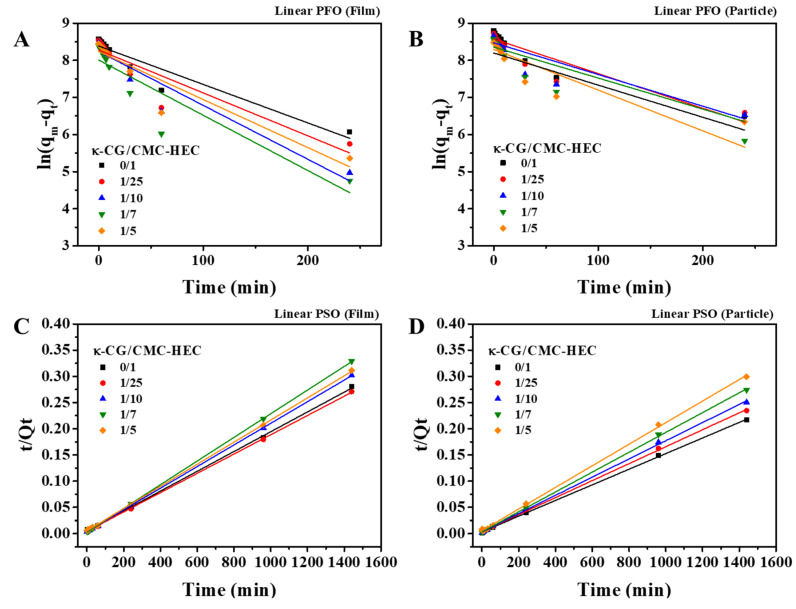
Fitting of the experimental absorption data in deionized water (pH 7) at 25 °C with the (**A**,**B**) linearized PFO and (**C**,**D**) PSO kinetic models.

The data of the absorption capacity versus time were further quantitatively analyzed by pseudo-first-order (PFO) and pseudo-second-order (PSO) kinetic models. The linearized PFO model (Equation (5)) exhibited a poor data fit with a relatively low correlation coefficient (R^2^) (Figure 7A,B), whereas the linearized PSO model (Equation (6)) exhibited a good data fit with a considerably high R^2^ (Figure 7C,D). Table 2 shows the absorption rate constants (k_1_, k_2_) and R^2^ calculated using both linearized kinetic models. The maximum absorption capacities calculated using the linearized PSO model were found to be more consistent with the measured values than those calculated using the linearized PFO model. However, as is well known, the isotherm or kinetic linearization equations may misinterpret the results [33]. In particular, it was recently emphasized that the linearized PFO model should not be ruled out because the values of q_m_ and R^2^ are worse than those obtained by the linearized PSO model, which induces erroneous conclusions [34]. This problem can be solved using the nonlinear forms of the PFO and PSO models [34]. 

**Figure 8:**
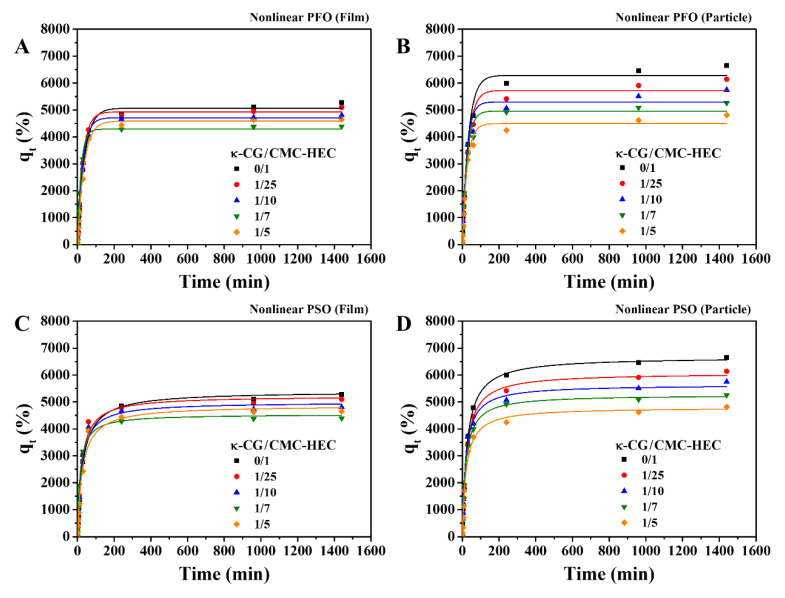
Effect of the absorption time on the water-absorption capacity of the hydrogel films and particles with different compositions in deionized water (pH 7) at 25 °C. The experimental data were fitted with the (**A**,**B**) nonlinear PFO and (**C**,**D**) PSO kinetic models.

The nonlinear forms of the kinetic models yielded the same results for the dried hydrogel particles. The water-absorption kinetics for the dried particles were better predicted by the nonlinear PSO model (Equation (8)) than the nonlinear PFO model (Equation (7)) (Figure 8 and Table 3). However, compared with the nonlinear PSO model, the nonlinear PFO model exhibited a better fit for the dried hydrogel film, with a higher R^2^ value. Furthermore, the maximum absorption capacities calculated by the nonlinear PFO model were closer to the experimental values than those obtained by the nonlinear PSO model (Table 3). The PSO model could represent the condition under which the adsorbent contains abundant active sites, and its kinetics are dominated by adsorption onto the active sites. The PFO model represents the condition under which a few active sites exist in an adsorbent material, and either external or internal diffusion is the rate-controlling step [35,36]. Compared with the gel particles, the large gel films have a small water interface, indicating the existence of a few active sites. Since the surface of the dried gel is an active site for water absorption, the particle sample has considerably more active sites than the film samples. In this case, the absorption onto the surface is the rate-controlling step. Compared with the gel particles, the gel films have considerably fewer active sites and a longer diffusion path, making the internal diffusion step the rate-controlling step for the water absorption of the gel film, which could be responsible for the good fit achieved with the nonlinear PFO kinetic model. Therefore, the nonlinear fit of the absorption kinetic model better describes the water absorption of the dried gel samples than the linearized model. For the particle sample, the absorption onto the surface is the rate-controlling step. 

**Figure 9:**
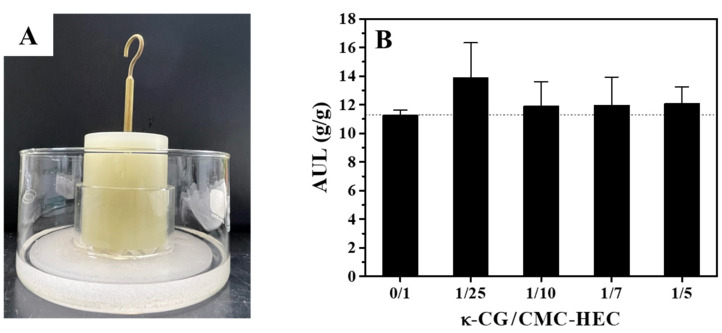
(**A**) Photographs of the AUL test equipment. (**B**) Effects of the κ-CG content on the AUL of the dried gel particles in deionized water (pH 7) at 25 °C.

The water absorbency under load (AUL) is another important evaluation parameter of SAPs, which could demonstrate their stability/strength [37,38,39]. The AUL of the dried gel samples was determined under an applied pressure of 0.3 psi, using an AUL equipment (Figure 9A). Interestingly, the AUL values of all the dried DN gel particles were higher than those of the dried CMC–HEC SN gel particles, which could be attributed to their improved mechanical properties (Figure 9B). In particular, the dried κ-CG/CMC–HEC (1/25) particles swelled to >13 times their own weight under load within 1 h, exhibiting the highest AUL, which can be attributed to their considerably high tensile strength. 

**Figure 10:**
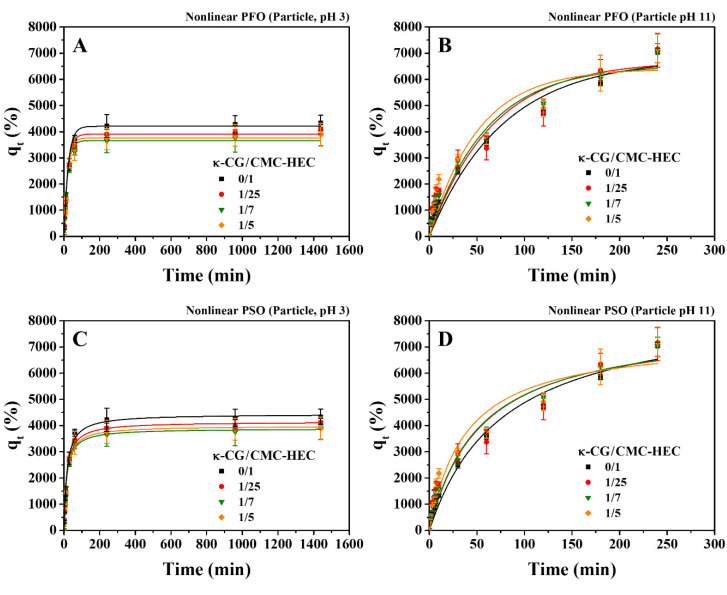
Effect of the absorption time on the water-absorption capacity of the hydrogel particles with different compositions in deionized water at (**A**,**C**) pH 3 and (**B**,**D**) pH 11 at 25 °C. The experimental data were fitted with the (**A**,**B**) nonlinear PFO and (**C**,**D**) PSO kinetic models.

To investigate the water-absorption behavior in response to pH values of the water, the dried gel particles were placed in water at pH 3.0 and 11, and the water-absorption capacities were measured as a function of the absorption time. Results showed that water-absorption capacities displayed a distinct pH dependence (Figure 10). When the pH of water was reduced from 7 to 3, the water-absorption capacities decreased (Figure 10A). The increased water-absorption capacities induced by increasing the pH of water to 11 have also been observed (Figure 10B). Similar pH-dependent swelling was observed for CA-crosslinked CMC–HEC hydrogels. Since the pK_a_ of the carboxylic acid in the polysaccharide is ~4.6, CA-crosslinked CMC–HEC hydrogels had a high degree of ionized carboxylic groups in alkaline pH [16]. The negatively charged CA crosslinked CMC network in the κ-CG/CMC–HEC DN or CMC–HEC SN gels may highly expand owing to electrostatic repulsion resulting in larger swelling in basic water than in neutral and acidic water. However, the DN gel became mechanically unstable and failed to maintain the gel particle after 4 h in the basic water. The data of the absorption capacity versus time were further quantitatively analyzed by nonlinear PFO and PSO kinetic models. The water-absorption kinetics for the dried particles in pH 3 and 11 water were better predicted by the nonlinear PSO model than the nonlinear PFO model (Figure 10 and Table 4).

## 3. Conclusions

In summary, we report a facile two-step method for synthesizing κ-CG/CMC–HEC DN hydrogels with excellent mechanical properties. The covalently crosslinked CMC–HEC hydrogels using the nontoxic and sustainable CA as the crosslinking agent were prepared first, after which they were immersed in hot aqueous solutions of κ-CG. Subsequently, the solutions were cooled, yielding fully biocompatible κ-CG/cellulose-derived DN gels. The combined covalent bonding and ionic crosslinks contributed to the high strength of the κ-CG/CMC–HEC hydrogels (tensile modulus: 0.202 MPa, tensile strength: 1.89 MPa, and fracture energy: 3.62 MJ/m^3^). The U_hys, n_/W_n_ values measured for the DN hydrogel during successive cyclic tensile loading–unloading tests highlighted the continuous fracturing of the sacrificial κ-CG network, which is responsible for the effective energy dissipation and the consequent improvement in the mechanical properties of DN gels. The water-absorption capacities and kinetics were dependent on the size of the dried hydrogels. The small gel particles exhibited a higher water-absorption capacity than the large films, attributed to their higher water interface area. The nonlinear PSO kinetic model better described the water absorption of the small gel particles, whereas the large gel film data were better fitted with the nonlinear PFO model. These observations imply that the internal diffusion could be the rate-controlling step for the large gel films because of their decreased water interface area and long internal diffusion path in the gel. The dried gels exhibited higher water uptake as the water became more alkaline. The dried DN gels exhibited a higher AUL than the dried SN gels, attributed to their improved mechanical properties. The proposed gels can be employed in various applications requiring environmentally benign superabsorbent gels with excellent mechanical properties.

## 4. Experimental Section

### 4.1. Materials 

CMC (DS = 0.89, M_w_ = 700,000), HEC (viscosity: 80–125 cP, 2% in H_2_O at 20 °C), κ-CG (#22,048), and CA were purchased from Sigma-Aldrich.

### 4.2. Preparation of DN Hydrogels

Figure 1 presents the schematic diagram for the preparation of the DN hydrogels. Firstly, HEC was dissolved in distilled water, after which CMC was added under vigorous mechanical stirring. The weight ratio of CMC to HEC was 3:1. Next, CA was added to the mixture solution of CMC and HEC. The resulting solution was poured into a glass petri dish, covered with filter paper, and left at 25 °C for 12 h to remove bubbles. The solution was dried at 40 °C for 48 h, after which it was kept at 80 °C for 24 h for the crosslinking reaction to occur, yielding the covalently crosslinked CMC–HEC films. The dried CMC–HEC films were swelled for 2 h in hot κ-CG solutions at 80 °C, confirming that no κ-CG solution was left. This means that all CGs and water have been successfully loaded onto the CMC/HEC gel. Afterward, they were cooled to 25 °C, affording κ-CG/CMC–HEC DN hydrogels. The total concentration was fixed at 14 wt%, but the κ-CG/CMC–HEC ratio was varied among 0:1, 1:25, 1:10, 1:7, and 1:5. The concentration of KCl was maintained at 6 wt% relative to that of κ-CG. The concentration of CA was maintained at 4 wt% (relative to the CMC–HEC ratio). If the crosslinking density is higher or lower than 4%, the soaking of CMC-HEC film in κ-CG solution led to very fast or slow water absorption resulting in non-uniform distribution of κ-CG in the CMC-HEC network.

### 4.3. Characterization

FTIR spectroscopy analysis was carried out to characterize the molecular structure of the CMC–HEC hydrogels before and after the crosslinking reaction with CA using a Bruker/Tensor27 spectrometer. The spectrum of the samples on the attenuated total reflectance plate was collected at 4 cm^−1^ resolution with 16 scans. The tensile mechanical properties of the hydrogels were characterized using a Universal Testing Machine (Instron 5543, Instron). The hydrogel samples were cut into dumbbell-shaped pieces with a gauge length of 9.53 mm, width of 3.18 mm, and thickness of 1 mm (ASTM D638 type V) for the tensile tests, with a crosshead speed of 50 mm/min and a static load of 1 kN. 

The tensile moduli (E) were obtained as the slope by linearly fitting the stress–strain curve in the initial stage. The toughness was calculated by the area enclosed by the stress–strain curve until the sample fractured. Five replications were run for each sample, and the average value ± standard deviation was adopted. In the tensile-hysteresis measurement, a dumbbell-shaped sample was first stretched to a predetermined strain ratio (ε_n_), after which it was unloaded to zero force at the same velocity of 50 mm/min. The cyclic hysteresis measurements were performed with multiple cycles of successive and progressive loading–unloading tests and no resting time between the consecutive loading cycles. The E values were calculated from the initial linear region of the stress–strain curves. Five replications were run for each sample, and the average value was employed to obtain reliable results. 

The morphology of the hydrogels was observed by scanning electron microscopy (JSM-6701F-JEOL Ltd., Tokyo, Japan) at an accelerating voltage of 15 kV. The gels were prepared by freeze-drying, followed by cryogenic fracturing in liquid nitrogen. The fractured surface was coated with a thin layer of Pt by sputtering before SEM observation. For the water-absorption measurement, the hydrogels were cut into 30 × 30 mm^2^ and 10 × 10 mm^2^ pieces, followed by immersion in ethanol for 48 h. Afterward, the dehydrated hydrogel was dried at 40 °C for mass stabilization. The absorption capacity was measured by dipping the dried hydrogels in deionized water at 25 °C. The pH of deionized water was adjusted to 3, 7, and 11 using HCl/NaOH to investigate the effects of pH on the water-absorption behavior of hydrogels. The water-swelled sample was removed from the liquid and gently wiped to remove the excess water, after which it was weighed. The absorption capacity (q_t_) at time t was calculated from the mass gain using the following equation:

(4) $$q_{t}(\%)=((W-W_{0})/W_{0})\times 100\%$$

where $W_{0}$ and W are the weights of the hydrogel’s initial and swollen masses, respectively.

The kinetics and mechanism of the water absorption were studied via the fitting of the absorption-capacity data to various models, including the linear and nonlinear forms of the PFO and PSO models. The mechanistic models are briefly described as follows [33,34,35,36]:

(5) $$\ln\left(q_{m}-q_{t}\right)=\ln\left(q_{m}\right)-k_{1}t,\;\mathrm{Linear}\;\mathrm{PFO}\;\mathrm{model}$$

(6) $$\frac{t}{q_{t}}=\frac{1}{q_{m}}t+\frac{1}{k_{2}q_{m}^{2}},\;\mathrm{Linear}\;\mathrm{PSO}\;\mathrm{model}$$

(7) $$q_{t}=q_{m}\left[1-\exp\left(-k_{1}t\right)\right],\;\mathrm{Nonlinear}\;\mathrm{PFO}\;\mathrm{model}$$

(8) $$q_{t}=\frac{k_{2}q_{m}^{2}t}{\left[k_{2}q_{m}t+1\right]},\;\mathrm{Nonlinear}\;\mathrm{PSO}\;\mathrm{model}$$

where q_m_ and q_t_ are the maximum absorption capacity and the absorption capacity at time t, respectively; k_1_ (min^−1^) and k_2_ (%^−1^·min^−1^) are the absorption rate constants of the PFO and PSO models, respectively.

The AUL was determined to measure the amount of fluid absorbed by the sample under a specific load. The bottom of a hollow glass cylinder (internal diameter and height of 6.0 and 4.0 cm, respectively) was sealed with a nylon fabric. The weighed dried gel (0.90 g) was evenly placed on the fabric surface at the bottom of the hollow cylinder, following which the cylinder was placed on sintered glass in a petri dish. A cylindrical solid load (d: 60 mm, applied pressure: 0.3 psi) was put on the dry samples in the glass cylinder. Thereafter, water (pH 7) was added when the liquid level was equal to the height of the sintered glass filter. After 60 min, the swollen particles were weighed again, and the AUL was determined using the following equation [37,38,39]:

(9) $$\mathrm{AUL}\;\left(g/g\right)=\frac{W-W_{0}}{W_{0}}$$

where $W_{0}$ and W are the weights of the dry and swollen gels, respectively.

## Figures and Tables

**Table 1:** Intensity ratio of the characteristic band associated with the crosslinking reaction of CMC–HEC via citric acid.

Sample	A_3383_/A_889_	A_1722_/A_889_	A_1230_/A_889_
CMC/HEC	0.61	0.20	0.26
CA-crosslinked CMC–HEC	0.37	7.45	0.39

**Table 2:** Kinetic-model parameters obtained using the linearized PFO and PSO models for the water absorption onto the dried gels in deionized water (pH 7) at 25 °C.

κ-CG/ CMC–HEC	Film							Particle						
	q_m_ (exp.)	PFO			PSO			q_m_ (exp.)	PFO			PSO		
		q_m_	k_1_	R^2^	q_m_	k_2_	R^2^		q_m_	k_1_	R^2^	q_m_	k_2_	R^2^
0/1	5277	4390	0.010	0.8967	5294	6.6 × 10^−6^	0.9996	6648	6149	0.021	0.8084	6711	6.2 × 10^−6^	0.9998
1/25	5097	3932	0.012	0.8364	5432	7.6 × 10^−6^	0.9998	6139	4784	0.086	0.7848	6177	6.8 × 10^−6^	0.9996
1/10	4808	3782	0.015	0.9106	4847	1.0 × 10^−5^	0.9999	5751	4260	0.008	0.7646	5760	8.5 × 10^−6^	0.9994
1/7	4393	3002	0.015	0.8298	4429	1.8 × 10^−5^	0.9999	5256	4000	0.011	0.6940	5291	9.9 × 10^−6^	0.9997
1/5	4649	3782	0.013	0.8791	4735	8.1 × 10^−6^	0.9997	4812	3630	0.009	0.7545	4857	7.5 × 10^−6^	0.9994

**Table 3:** Kinetic-model parameters obtained using the nonlinear PFO and PSO models for the water absorption onto the dried gels in deionized water (pH 7) at 25 °C.

κ-CG/ CMC–HEC	Film							Particle						
	q_m_ (exp.)	PFO			PSO			q_m_ (exp.)	PFO			PSO		
		q_m_	k_1_	R^2^	q_m_	k_2_	R^2^		q_m_	k_1_	R^2^	q_m_	k_2_	R^2^
0/1	5277	5067	0.027	0.9971	5400	6.4 × 10^−6^	0.9956	6648	6284	0.030	0.9860	6673	6.2 × 10^−6^	0.9982
1/25	5097	4929	0.034	0.9978	5228	8.8 × 10^−6^	0.9924	6139	5727	0.033	0.9818	6078	7.3 × 10^−6^	0.9982
1/10	4808	4712	0.035	0.9990	4994	9.5 × 10^−6^	0.9922	5751	5297	0.041	0.9731	5638	9.6 × 10^−6^	0.9924
1/7	4393	4288	0.052	0.9954	4545	1.5 × 10^−5^	0.9938	5256	4960	0.040	0.9842	5267	1.0 × 10^−5^	0.9986
1/5	4649	4586	0.029	0.9967	4871	7.8 × 10^−6^	0.9876	4812	4494	0.038	0.9872	4796	1.0× 10^−5^	0.9905

**Table 4:** Kinetic-model parameters obtained using the nonlinear PFO and PSO models for the water absorption onto the dried gels in deionized water (pH 3 and 11) at 25 °C.

κ-CG/ CMC–HEC	Particle (pH 3)							Particle (pH 11)						
	q_m_ (exp.)	PFO			PSO			q_m_ (exp.)	PFO			PSO		
		q_m_	k_1_	R^2^	q_m_	k_2_	R^2^		q_m_	k_1_	R^2^	q_m_	k_2_	R^2^
0/1	4335	4205	0.043	0.9808	4437	1.4 × 10^−5^	0.9908	7079	6798	0.013	0.9641	8643	1.5 × 10^−6^	0.9766
1/25	4100	3911	0.047	0.9864	4141	1.5 × 10^−5^	0.9955	7113	6698	0.015	0.9090	7896	2.4 × 10^−6^	0.9351
1/7	3898	3659	0.050	0.9843	3885	1.8 × 10^−5^	0.9988	7009	6535	0.017	0.9526	7962	2.3 × 10^−6^	0.9705
1/5	3884	3761	0.049	0.9835	3983	1.7 × 10^−5^	0.9889	7178	6405	0.020	0.9033	7385	3.6 × 10^−6^	0.9389
